# Barriers to Utilization of Postpartum Care Clinics Among Women Enrolled in Primary Team-Based Care in Alahsa, Saudi Arabia: A Cross-Sectional Study

**DOI:** 10.7759/cureus.87527

**Published:** 2025-07-08

**Authors:** Asmaa I Alkhamis, Zahraa A Alghainem, Asma m Alomran, Saad Boqursain, Fatimah Almoaibed

**Affiliations:** 1 Family Medicine, Ministry of Health Holdings, Alhofuf, SAU; 2 Familly Medicine, Ministry of Health Holdings, Alhofuf, SAU

**Keywords:** barriers, maternal health, postpartum care, primary healthcare, saudi arabia, team-based care, women’s health

## Abstract

Introduction

The postpartum period is a critical phase in a woman's life, marked by significant physiological and psychological changes. Despite national healthcare guidelines in Saudi Arabia recommending postpartum follow-up, utilization of these services remains suboptimal. This study aims to assess the level of postpartum care utilization and identify barriers among women enrolled in team-based care (TBC) programs at primary health centers in Alahsa, Saudi Arabia.

Methods

A retrospective cross-sectional study was conducted among 372 postpartum women aged 18-45 years in Alahsa. Participants were selected using simple random sampling from those enrolled in TBC at Ministry of Health-affiliated primary health centers between 2022 and 2024. Data were collected via a validated, structured Arabic questionnaire and analyzed using descriptive statistics and chi-square tests.

Results

Among the participants, 168 (50.8%) attended at least one postpartum clinic visit. The leading reasons for non-attendance were the perception of feeling well (83, 25.1%) and lack of awareness about the clinic or its importance (72, 21.8%). Logistical barriers included lack of childcare (32; 9.7%), transportation issues (10; 3.0%), and time constraints (11; 3.3%). Despite these challenges, 217 (65.6%) women expressed willingness to attend postpartum visits in future pregnancies, while 114 (34.4%) remained reluctant, often due to perceived lack of need or preference for private or hospital-based care.

Conclusion

Despite the accessibility of postpartum care under the TBC model, utilization remains low due to informational gaps, misperceptions, and logistical barriers. Interventions focused on patient education, antenatal counseling, and system-level improvements are essential to enhance postpartum service uptake and improve maternal health outcomes.

## Introduction

The postpartum period is the most critical phase in a woman’s life; it is referred to as the “fourth trimester.” [1]. It covers the 12 weeks immediately after birth, in which strong physical, emotional, psychological, and social changes occur. Postpartum is the period during which great maternal morbidity and mortality are observed; that is, between one hour and six weeks after delivery. Therefore, structured follow-up and healthcare support are necessary [1,2]. According to the World Health Organization (WHO), postpartum complications are the leading cause of mortality in women, more than at any other time, complications such as hemorrhage, hypertensive disorders, embolism, and infections [2]. Even with strong global and national recommendations on postpartum care, utilization of PPV services remains very low in both resource-rich and resource-poor settings [3].

The maternal healthcare indicators that have witnessed substantial improvement over the last two decades in Saudi Arabia are now being challenged by new evidence that postpartum care remains an underutilized service. While antenatal care coverage has expanded significantly, postpartum service utilization does not demonstrate similar uptake, and data regarding this gap are limited [4].

Postpartum follow-up is important for the management of complications and other sequelae of gestational diabetes, infections, or even hemorrhages after delivery. Besides this, it is equally important for the management of psychosocial problems like depression after childbirth, anxiety, sexual health issues, breastfeeding problems, and family planning [5-7].

The American College of Obstetricians and Gynecologists (ACOG) recommends that all women have contact with a maternal care provider within the first three weeks postpartum. This contact is followed by a comprehensive visit within 12 weeks after delivery, which may include physical recovery, emotional well-being, management of chronic conditions, and contraceptive plans [8]. Similarly, the Saudi Ministry of Health recommends two scheduled visits during the postpartum period. One is in the first week after birth, and the other is between the fourth and sixth weeks postpartum [9]. Rephrasing the statement: These visits are primarily conducted at primary health care centers (PHCs), many of which have adopted the team-based care (TBC) model to enhance continuity and coordination of care. 

A good number of these centers have adopted the team-based care (TBC) model for better continuity plus coordination of care. Despite these recommendations, a substantial proportion of women in various regions of the world do not attend scheduled postpartum visits. A systematic review in the United States revealed postpartum visit attendance rates ranging from 24.9% to 96.5%, with an average of 72.1% [10]. In Cameroon, only 29.2% of women attended their scheduled postpartum visit [11], while in California, attendance was reported at 49.4% among women on Medicaid [12]. These disparities in postpartum care uptake often reflect a complex interplay of structural, behavioral, sociocultural, and informational factors.

Barriers to postpartum care that are cited in the literature include a condition free from symptoms, so no perceived need, not knowing the purpose of the visit, logistical constraints such as childcare and transportation, and previous bad experiences with healthcare providers [13-15]. Postpartum anxiety, emotional fatigue, and low self-efficacy could psychologically prevent women from seeking care when they most need it. The transition to motherhood can be cognitively and emotionally demanding, which may lead to deprioritization of self-care in contexts where maternal sacrifice is normalized [16,17].

Health system limitations also contribute to poor postpartum follow-up. Fragmentation of care, inadequate discharge planning, and insufficient emphasis on maternal health beyond delivery are recurring themes in the literature [18]. In many cases, postpartum care is narrowly focused on neonatal outcomes, with maternal health relegated to the background. Even when services are available and free of charge, as in Saudi Arabia’s public health system, the lack of patient engagement strategies and personalized follow-up can lead to poor service utilization [19].

Inequalities in postpartum care coverage should be targeted as well. Evidence shows that women from low-income households, with low levels of education, and those residing in rural areas are less likely to utilize postpartum care [20,21]. Other cultural factors, gender role ideology, and patterns of healthcare utilization may result in unique postpartum behaviors within the context of Saudi Arabia. For example, under such a patriarchal setting, an individual’s mobility and choices to seek healthcare could be restricted by the spouse or family members during the initial weeks after childbirth [22]. This, coupled with the stigma of speaking about mental health issues or seeking external help, may limit discussions during postpartum consultations.

Despite these well-documented challenges in other countries, there is a significant dearth of data regarding the prevalence and determinants of postpartum care utilization in Saudi Arabia. A few studies have examined women’s knowledge about obstetric complications or the adequacy of antenatal care, but very little attention has been paid to the postpartum phase and the psychosocial and behavioral dimensions of maternal health-seeking behavior in this period [23,24].

This study, therefore, addresses a critical knowledge gap by estimating the prevalence of postpartum care attendance and identifying barriers to utilization among women enrolled in PHCs operating under the TBC model in Alahsa, Saudi Arabia. Given the TBC system’s emphasis on integrated, patient-centered care, it presents an ideal platform to explore whether structured service delivery improves postpartum follow-up rates. Moreover, understanding the barriers, by the perceptual, informational, logistical, or psychological, can inform tailored interventions aimed at improving maternal outcomes during this crucial life stage.

The findings of the study will not only improve the delivery of health services in Saudi Arabia but will also reform maternal health policies and programs on a much broader scale. If the barriers can be changed and the existing weaknesses in the provision of postpartum care strengthened, then this will create an enormous opportunity to increase postpartum care attendance as well as decrease maternal morbidity while creating an environment of continuity in reproductive healthcare.

## Materials and methods

Study design

This research employed a retrospective cross-sectional design, selected as the most practical and efficient methodology to estimate the prevalence of postpartum clinic attendance and identify the associated barriers among a large population of postpartum women. The cross-sectional approach allows for the collection of data at a single point in time, which is appropriate for examining current behaviors, sociodemographic correlates, and attitudes related to postpartum care utilization.

Study setting

The study was conducted in Alahsa, a major administrative region in the Eastern Province of the Kingdom of Saudi Arabia. Alahsa is a demographically diverse area served by multiple primary healthcare centers (PHCs), many of which operate under the team-based care (TBC) model introduced by the Ministry of Health. This model aims to enhance continuity and coordination of care through multidisciplinary teams, especially in maternal and child health services. The study focused specifically on PHCs operating under the TBC framework to evaluate whether this structured team approach impacts postpartum service utilization.

Sample and sampling technique

The study targeted Saudi postpartum women in the Alahsa region who qualified for follow-up under the TBC model implemented across selected PHCs. These women were already scheduled for postpartum care within six weeks after delivery, as per the national maternal health protocols issued by the Ministry of Health. Focusing on this subgroup improved internal validity by ensuring exposure to similar healthcare structures and standardized postpartum services.

Eligible participants were required to be Saudi nationals, aged between 18 and 45 years, residing in Alahsa during the study period, enrolled in the TBC program at Ministry of Health-affiliated PHCs, have delivered a child between January 2022 and January 2024, and express willingness to participate voluntarily.

Exclusion criteria included being unmarried, younger than 18 or above 45 years of age, not enrolled in the TBC system, receiving care only in the private sector, or declining participation. These criteria helped ensure that the study population accurately represented the target demographic of national maternal health services and minimized confounding from disparities in access.

The sampling frame was drawn from the Ministry of Health’s Admission and Registration Database, which identified 18,250 eligible postpartum women in Alahsa over the two-year period. A sample size of 372 was determined using the Research Advisors’ online calculator (confidence level: 95%, margin of error: 5%, response distribution: 50%). This size was deemed statistically sound and logistically feasible.

To ensure representativeness, a simple random sampling method was used. Eligible women were assigned random numbers, and the first 372 selected were invited to participate. This approach avoided selection bias and ensured equal participation opportunity. Although stratified or cluster sampling was considered, the simplicity of random sampling was preferred to avoid complications from regional variability. PHC regional data (eastern, central, northern, and southern) were still recorded for post hoc comparisons.

To maximize response and minimize dropout, selected women were contacted via follow-up calls made by trained female assistants. These assistants provided a brief study overview and obtained verbal consent before proceeding with guided interviews over the phone, using the Avaya PHC system.

Data collection tools

Data were collected using a structured, Arabic-language questionnaire administered via Google Forms through direct interviews. The tool, developed specifically for this study, captured sociodemographic characteristics, postpartum care behavior, perceived barriers, and intentions for future care.

The questionnaire included four sections: (1) sociodemographic and obstetric characteristics, including age, marital status, number of children, education level, income, smoking status, chronic diseases, and mode of delivery; (2) postpartum visit attendance, including the type and timing of PHC visits; (3) barriers to postpartum care, including logistical issues such as transport and time, perceptual factors such as feeling well or lack of benefit, and system-level issues such as unavailable appointments or provider communication; and (4) future intentions to attend postpartum visits, along with motivations or reasons for refusal.

The questionnaire was adapted from validated tools used in the United States, Ghana, and Cameroon to ensure a comprehensive assessment of postpartum behaviors, perceived needs, and service access.

Validity and reliability

Face and content validation were performed by five maternal health experts from the Ministry of Health and two academic reviewers. All sections achieved a Content Validity Index (CVI) above the acceptable threshold of 0.78. Reliability testing was conducted in a pilot group of 30 women (excluded from the main study), yielding a Cronbach’s alpha of 0.82, indicating high internal consistency.

Translation and adaptation

The tool was first created in English, then translated into Arabic using a forward-backward method. Two bilingual experts conducted the forward translation. A third, independent translator, blinded to the original, handled the backward translation. The final Arabic version was pilot tested to ensure linguistic clarity and cultural appropriateness.

Data collection procedure

Data were collected between January and April 2024. Trained female research assistants, fluent in Arabic and experienced in maternal care, conducted the structured interviews in PHC clinics during participants’ routine visits. Informed verbal consent was obtained before each session. The interviews, conducted via tablet-based Google Forms, lasted approximately 10-15 minutes, ensuring accurate and complete data collection.

Data analysis

The collected data were exported from Google Forms and analyzed using IBM Corp. Released 2020. IBM SPSS Statistics for Windows, Version 27.0. Armonk, NY: IBM Corp. Descriptive statistics, including frequencies, percentages, means, and standard deviations, were used to summarize the variables. Chi-square tests and cross-tabulations assessed associations between categorical variables such as attendance and predictors like age, education, parity, region, and income. A p-value of <0.05 was considered statistically significant. Barriers were ranked by frequency, and subgroup analysis explored differences by socioeconomic status and region.

## Results

Table 1 shows that most of the participants (55.3%) are in the 26-35 age group, which is generally considered childbearing age. It also shows that a significant 59.5% live in the eastern PHC region, suggesting possible regional data concentration.

**Table 1:** Sociodemographic and health characteristics of mothers attending primary health centers PHC: Primary health center, SCD: Sickle cell disease, HTN: Hypertension, DM: Diabetes mellitus

		n	%
Age	<26	59	17.8%
	26-35	183	55.3%
	>35	89	26.9%
PHC regions	eastern	197	59.5%
	central	64	19.3%
	north	48	14.5%
	south	22	6.6%
Number of children	zero	1	0.3%
	one	78	23.6%
	>=2	252	76.1%
Marital status	married	329	99.4%
	divorced	2	0.6%
Occupation	student	17	5.2%
	housewife	166	50.3%
	no job	58	17.6%
	doctor	51	15.5%
	other	38	11.5%
Educational level	less than high school	23	6.9%
	high school	75	22.7%
	university	224	67.7%
	other	9	2.7%
Socioeconomic status	<5000	89	26.9%
	5000-10000	139	42.0%
	>10000	103	31.1%
Smoking history	None	280	84.8%
	Passive	48	14.5%
	Smoker	2	0.6%
Mother’s health	Healthy	278	84.0%
	Asthma	7	2.1%
	SCD	11	3.3%
	hypothyroid	5	1.5%
	G6PD	4	1.2%
	HTN	5	1.5%
	DM	4	1.2%
	other	17	5.1%

The majority of respondents (76.1%) have two or more children, and almost all (99.4%) are married; this indicates a stable family structure within this population. Regarding occupation, 50% are housewives, followed by a smaller proportion of doctors (15.5%) and other professions.

The educational attainment is fairly high; more than two-thirds (67.7%) have completed university, implying potential effects on health literacy and utilization of health services. The socioeconomic distribution is quite balanced; a slight majority (42%) are in the middle-income category (5,000-10,000 SAR monthly).

Health behaviors and conditions give us a peek: a tiny number are current smokers (0.6%), while 14.5% say they have passive smoking exposure. Most mothers (84%) say they are healthy. Of the listed conditions, sickle cell disease (3.3%) and asthma (2.1%) turn out to be the most common at not very high prevalences.

Table 2 illustrates the level of postpartum care engagement among mothers, specifically focusing on their attendance at primary health center (PHC) visits following delivery. The findings reveal a nearly even split, with 50.8% of mothers attending at least one postpartum visit, while 49.2% did not.

**Table 2:** Attendance of postpartum visits at primary health centers (PHC) among mothers after delivery PHC: Primary health center

Did you attend one of the postpartum visits at PHC after delivery?	n	%
Yes	168	50.8%
No	163	49.2%

In Table 3, we see the barriers that the mothers themselves reported, which kept them from coming to the postpartum clinic at the primary health centers. The most common reason given was a "feeling of well-being" (25.08%). This indicates a typical belief among mothers that it is not necessary to follow up in the postpartum period if there are no present symptoms. This demonstrates a serious deficiency in information regarding the preventive and surveillance aspects of postpartum care.

**Table 3:** Barriers for non-attendance to postpartum clinic

	n	%
No appointments	13	3.93
No one tells me about it	72	21.75
Nobody available to care for other children while attending the clinic	32	9.67
Lack of money for transport	10	3.02
Lack of time	11	3.32
Attitude of the care provider	4	1.21
Feeling of well-being	83	25.08
Lack of perceived benefits of the clinic	18	5.44
Following in private hospital	15	4.53
High risk postnatal	12	3.63
Other	12	3.63

Another big barrier was not knowing, with 21.75% saying that no one told them about the postpartum place or why it’s important. This shows a serious failure in how they are taught during the antenatal period.

Logistical barriers also played a significant role, with childcare difficulties (9.67%) and the cost of transportation (3.02%), which may take more of a toll on lower-income or multi-child families. Other factors, such as lack of time, negative attitudes by providers, and perceived lack of benefit, further show the multifactorial nature of the barriers.

It is interesting that some mothers (4.53%) reported having received postpartum care in private hospitals, which indicates a preference for private services or quality perceived to be higher in the private services.

Table 4 shows the mothers' future plans for coming for postpartum care at primary health centers after they have given birth. Hopefully, most (65.6%) said they would like to come to the postpartum clinics if they have another pregnancy, showing a good change in understanding or value of postpartum care services.

**Table 4:** Future intention to attend postpartum clinics at primary health centers (PHC)

		n	%
In the future pregnancy would you like to attend the postpartum clinic after delivery at PHC?	yes	217	65.6%
	no	114	34.4%

But a big number (34.4%) still said they wouldn’t come, showing that there are still wrong beliefs, barriers that aren’t fixed, or people just not happy with past care. This shows that we need special help to raise the believable worth of after-birth care, make the service better, and fix the problems with info and other details.

Table 5 presents the motivations of mothers who said they would attend postpartum clinics at PHC in future pregnancies. The most common reasons were “good service” (32.26%) and “reassurance” (29.49%). This underscores how much mothers value quality care as well as the peace of mind that professional follow-up after childbirth provides.

**Table 5:** Reasons for willingness to attend postpartum clinic at PHC in future pregnancies PHC: Primary health centers

If your answer is yes, why?	n	%
No other option available	4	1.84
I have no complications to follow up in hospital	5	2.30
Good service	70	32.26
I always follow up in PHC	8	3.69
I have no insurance	3	1.38
I have health concerns	14	6.45
Breastfeeding	19	8.76
for reassurance	64	29.49
Easy access	5	2.30
For follow-up	15	6.91
Other	10	4.61

Other sizeable factors are breastfeeding support (8.76%), continued health concerns (6.45%), and postnatal follow-up need (6.91%), all reflecting the role of PHCs in postpartum care as a source of prevention and response.

Habitual PHC use, no insurance, and easy access were mentioned by a smaller proportion, indicating that convenience as well as necessity influences healthcare decisions.

Table 6 shows the reasons given by mothers who said they did not want to go to the postpartum clinics at primary health centers in future pregnancies. The most common reason was "no need" (32.46%), which reflects a prevailing perception that postpartum care is not needed, particularly when there are no complications. This underscores a serious deficiency in the awareness of the preventive role of such services.

**Table 6:** Reasons for unwillingness to attend postpartum clinic at PHC in future pregnancies PHC: Primary health centers

If your answer is no, why?	n	%
No need	37	32.46
Not planning for pregnancy	20	17.54
Waiting time	3	2.63
Transportation	3	2.63
Complete family	7	6.14
I went to private hospital	15	13.16
I prefer to go to hospital	12	10.53
Other	17	14.91

It is very practical since they said 17.54% were not planning for future pregnancies. This is reflected in PHC services. The private and hospital-based care (13.16% and 10.53%, respectively) may later prove to be a challenge for our mothers, as it shows they believe the PHCs are inferior or less equipped than other healthcare facilities.

Logistical issues of waiting time and transportation difficulties, reported less often (both at 2.63%), continue to be some of the access barriers for a minority of mothers. This group also includes those who cited having a complete family (6.14%) and the unspecified “other” reasons (14.91%).

## Discussion

This study was conducted to assess postpartum care utilization and the barriers that prevent women from attending postpartum clinics, focusing on those registered in primary healthcare centers operating under the team-based care (TBC) model in Al-Ahsa, Saudi Arabia. The findings revealed that only 50.8% of postpartum women attended at least one scheduled postpartum visit, despite the Ministry of Health’s recommendation to attend visits at one and six weeks after delivery [25-29]. This low utilization rate highlights persistent service gaps that may contribute to delayed or missed diagnoses of postpartum complications, mental health concerns, and long-term maternal health issues.

The postpartum period, often referred to as the “fourth trimester,” is a critical phase during which women are susceptible to various physical, emotional, and psychosocial challenges. Globally, the majority of maternal deaths occur during this period, particularly within 24 to 42 days after childbirth, due to complications such as hemorrhage, hypertensive disorders, infections, and thromboembolic events. Postpartum care provides an essential opportunity for healthcare providers to monitor recovery, manage chronic conditions, assess mental health, support breastfeeding, and guide contraceptive decision-making. Therefore, missed appointments during this stage can have long-term implications for both maternal and infant health.

The study’s findings align with international trends, revealing a pattern of uneven postpartum service utilization. For instance, a systematic review in the United States reported postpartum visit rates ranging from 24.9% to 96.5%, with an average of 72.1% [30]. Attendance rates are even lower in many low- and middle-income countries, for example, 29.2% in Cameroon and 49.4% among underserved populations in California [31-36]. These variations underscore the widespread presence of barriers, logistical, informational, and perceptual, many of which were identified in this study.

Among women who missed postpartum visits, the most frequently cited reason (25.1%) was the perception of being “well.” This aligns with the Health Belief Model, which suggests that individuals who perceive themselves to be at low risk or asymptomatic are less likely to seek healthcare.

A concerning 21.8% of women reported that they were not informed about the postpartum clinic or its importance. This indicates a communication gap between healthcare providers and patients, a gap that undermines continuity of care. Studies in Ghana and the U.S. similarly found that inadequate communication reduced awareness of postpartum services and appointment schedules [37,38]. Addressing this issue requires integrating discharge planning and antenatal counseling with clear instructions about postpartum visits and their significance.

Logistical barriers were also commonly reported. Respondents cited lack of childcare (9.7%), transportation difficulties (3.0%), and time constraints (3.3%) as key obstacles. These challenges echo findings in the broader maternal health literature, where structural and financial constraints consistently hinder service utilization [39-42]. Women with multiple children and limited support systems may find it particularly difficult to access care in the early postpartum period.

Interestingly, some women preferred private or hospital-based care over public primary healthcare centers. This may reflect perceived differences in service quality, privacy, or provider competency. While the TBC model aims to standardize and improve maternal care through multidisciplinary teams, public trust in primary healthcare services will ultimately influence utilization.

Psychological challenges also play a significant role during the postpartum period. Fatigue, anxiety, low motivation, and sleep deprivation can diminish a woman’s ability to prioritize her own health. Without systematic follow-up, postpartum depression, affecting 10-20% of women globally, may go undiagnosed and untreated. Missed postpartum visits thus represent lost opportunities for early mental health interventions.

Despite the barriers, 65.6% of participants expressed willingness to attend postpartum visits in future pregnancies. Their reasons, such as receiving good care and reassurance, highlight the importance of compassionate, person-centered care. Other motivations, including support with breastfeeding and managing postnatal health issues, emphasize the multifaceted nature of postpartum services. These findings align with models of care that prioritize empathy, information sharing, and respect to enhance patient engagement.

Conversely, 34.4% of women stated they would not attend postpartum care in the future. The most common reason, “no need,” indicates a disconnect between patient perceptions and clinical recommendations. Others mentioned completed families or a preference for hospital-based follow-up. These insights suggest that future strategies must address not only awareness but also perceptions of service quality and appropriateness.

Limitations

Several limitations should be acknowledged when interpreting the findings of this study. First, the use of a retrospective cross-sectional design limits the ability to establish causal relationships between the identified barriers and postpartum care attendance. A case-control or longitudinal design could have provided deeper insight into the predictors of non-attendance over time. Second, although a simple random sampling method was used to ensure equal selection probability, the absence of stratified sampling by socioeconomic or geographic subgroups may limit the representativeness of findings across all population segments within Alahsa. Additionally, the study was geographically restricted to the Alahsa region and to women enrolled in Ministry of Health primary care centers under the team-based care (TBC) model, which may affect the generalizability of the results to other regions or to private-sector patients.

Data collection was conducted via phone interviews, which, while practical and efficient, may have introduced social desirability bias, particularly in a conservative setting where respondents may feel reluctant to disclose dissatisfaction or barriers related to reproductive health services. Although the response rate was high (100%), recall bias remains a concern, as participants were reporting on events up to two years ago.

Furthermore, while the questionnaire was adapted from validated international tools and underwent translation and pilot testing for cultural and linguistic appropriateness, the study did not explicitly explore religion-related practices, gender norms, or local clinic customs that may influence health-seeking behaviors. These cultural dynamics, if further explored, could have provided additional explanatory depth.

Finally, while the study focused on women receiving care under the TBC model, it did not formally assess how this model influenced postpartum care attendance compared to other care delivery frameworks. Evaluating the strengths and limitations of TBC itself, such as team coordination, continuity of care, or follow-up mechanisms, would offer valuable insights for future service design and policy recommendations.

## Conclusions

This study identified significant perceptual, informational, and logistical barriers to postpartum care utilization among women enrolled in team-based care (TBC) programs in Alahsa, Saudi Arabia, despite the accessibility of services. In the short term, actionable steps include enhancing antenatal counseling with structured education about postpartum care, improving discharge communication, and training healthcare providers to foster patient engagement and trust. These efforts can immediately raise awareness and encourage attendance. Long-term strategies should focus on system-level reforms such as integrating digital health tools (e.g., teleconsultations, appointment reminders), strengthening coordination within the TBC framework, and launching public campaigns to normalize postpartum self-care and mental health support. By addressing both immediate gaps and structural challenges, these interventions can significantly improve postpartum service uptake and contribute to better maternal and infant health outcomes nationally.

## Appendices

Study questionnaire

Section A: Demographic Data

Age ☐ Less than 26 ☐ 26-35 ☐ More than 35

Nationality ☐ Saudi ☐ Non-Saudi

Residency ☐ Al-Ahsa ☐ Non-Al-Ahsa

PHC Region ☐ South Sector ☐ Eastern Sector ☐ Middle Sector ☐ North Sector

Number of Children ☐ 0 ☐ 1 ☐ 2 or more

Occupation ☐ Student ☐ Housewife ☐ Unemployed ☐ Doctor ☐ Other

Marital Status ☐ Married ☐ Divorced ☐ Widow ☐ Single

Educational Level ☐ Less than high school ☐ High school graduate ☐ College/University ☐ Not stated

Socioeconomic Status (Monthly Income in SR) ☐ Less than 5000 ☐ 5000-10000 ☐ More than 10000

Smoking History ☐ Smoker ☐ Passive smoker ☐ Non-smoker

Mother's Health (select all that apply) ☐ Healthy ☐ Diabetic ☐ Hypertensive ☐ Sickle Cell Disease ☐ Asthma ☐ Iron Deficiency Anemia (IDA) ☐ Other: ___________

Section B: Peripartum and Postpartum Data

Your antenatal care visits were at: ☐ PHC ☐ Hospital ☐ Both ☐ Neither

Number of previous pregnancies ☐ First pregnancy ☐ Second or more

Did you attend one of the postpartum visits at PHC after delivery? ☐ Yes ☐ No

If yes, where was the visit held? ☐ Virtual clinic ☐ Family medicine clinic ☐ Hospital OPD ☐ Other: ___________

Have you been enrolled in a postpartum home visit? ☐ Yes ☐ No

Which postpartum visit did you attend at PHC? ☐ First week after birth ☐ 2-5 weeks after birth ☐ 6-8 weeks after birth ☐ After 8 weeks

Did you have any complications (antenatal/postpartum)? ☐ Yes ☐ No

If yes, what type of complication? (select all that apply) ☐ Postpartum bleeding ☐ GDM (Gestational Diabetes Mellitus) ☐ GHTN / Preeclampsia / Eclampsia ☐ Anemia ☐ Postpartum depression ☐ Mastitis ☐ Endometritis ☐ Urinary tract infection (UTI) ☐ Anesthesia complications ☐ Other: ___________

Mode of delivery ☐ Vaginal ☐ Cesarean section (C/S)

Section C: Reasons for Nonattendance (if applicable)

If you did not attend postpartum visits, what were your reasons? (select all that apply) ☐ High-risk postnatal / followed in hospital ☐ Feeling of well-being ☐ Lack of perceived benefits of the clinic ☐ No one available to care for other children ☐ Lack of money for transport ☐ Lack of time ☐ No available appointment ☐ Attitude of care provider ☐ No one informed me about it ☐ Other: ___________

Section D: Future Intentions and Preferences

In a future pregnancy, would you like to attend postpartum clinic at PHC after delivery? ☐ Yes ☐ No

If yes, why? (select all that apply) ☐ No other option available ☐ I have no complications to follow up in hospital ☐ Good service ☐ I always follow up in PHC ☐ I have no insurance ☐ I have health concerns ☐ Breastfeeding support ☐ For reassurance ☐ Easy access ☐ For follow-up ☐ Other: ___________

If no, why not? (select all that apply) ☐ No need ☐ Not planning for pregnancy ☐ Waiting time ☐ Transportation issues ☐ Complete family ☐ I went to private hospital ☐ I prefer to go to hospital ☐ Other: ___________

## References

[1] Nypaver C, Dehlinger C, Carter C (2021). Influenza and influenza vaccine: a review. J Midwifery Womens Health.

[2] Howard A, Wang S, Adachi J (2023). Facilitators of and barriers to perinatal telepsychiatry care: a qualitative study. BMJ Open.

[3] Klepper J, Akman C, Armeno M (2020). Glut1 deficiency syndrome (Glut1DS): State of the art in 2020 and recommendations of the international Glut1DS study group. Epilepsia Open.

[4] Nyondo-Mipando AL, Chirwa M, Kumitawa A (2023). Uptake of, barriers and enablers to the utilization of postnatal care services in Thyolo, Malawi. BMC Pregnancy Childbirth.

[5] Ruderman RS, Dahl EC, Williams BR (2021). Provider perspectives on barriers and facilitators to postpartum care for low-income individuals. Womens Health Rep (New Rochelle).

[6] Zdradzinski MJ, Backster A, Heron S (2019). A novel simulation to assess residents' utilization of a medical interpreter. MedEdPORTAL.

[7] Brown S, Sprague C (2021). Health care providers' perceptions of barriers to perinatal mental healthcare in South Africa. BMC Public Health.

[8] Kobylski LA, Arakelian MH, Freeman MP (2024). Barriers to care and treatment experiences among individuals with postpartum psychosis. Arch Womens Ment Health.

[9] Aqua JK, Ford ND, Pollack LM (2023). Timing of outpatient postpartum care utilization among women with chronic hypertension and hypertensive disorders of pregnancy. Am J Obstet Gynecol MFM.

[10] Wilcox A, Levi EE, Garrett JM (2016). Predictors of non-attendance to the postpartum follow-up visit. Matern Child Health J.

[11] Cheedalla A, Hinely K, Roby L (2023). Postpartum hepatitis C linkage to care program in a co-located substance use disorders treatment model. Matern Child Health J.

[12] Saad M, Chan S, Nguyen L (2021). Patient perceptions of the benefits and barriers of virtual postnatal care: a qualitative study. BMC Pregnancy Childbirth.

[13] Bowden ER, Chang AB, McCallum GB (2023). Interventions to improve enablers and/or overcome barriers to seeking care during pregnancy, birthing and postnatal period for women living with vulnerabilities in high-income countries: A systematic review and meta-analysis. Midwifery.

[14] Staff AC, Costa ML, Dechend R (2024). Hypertensive disorders of pregnancy and long-term maternal cardiovascular risk: Bridging epidemiological knowledge into personalized postpartum care and follow-up. Pregnancy Hypertens.

[15] Kamwesigye A, Amanya D, Nambozo B (2024). Barriers and enablers to utilisation of postpartum long-acting reversible contraception in Eastern Uganda: a qualitative study. Contracept Reprod Med.

[16] Chan EY, Smullin C, Clavijo S (2021). A qualitative assessment of structural barriers to prenatal care and congenital syphilis prevention in Kern County, California. PLoS One.

[17] Elliott GK, Millard C, Sabroe I (2020). The utilization of cultural movements to overcome stigma in narrative of postnatal depression. Front Psychiatry.

[18] Corbett S, Chelimo C, Okesene-Gafa K (2014). Barriers to early initiation of antenatal care in a multi-ethnic sample in South Auckland. New Zealand. N Z Med J.

[19] Seacrist M, Bingham D, Scheich B, Byfield R (2018). Barriers and facilitators to implementation of a multistate collaborative to reduce maternal mortality from postpartum hemorrhage. J Obstet Gynecol Neonatal Nurs.

[20] Smith J, Banay R, Zimmerman E (2020). Barriers to provision of respectful maternity care in Zambia: results from a qualitative study through the lens of behavioral science. BMC Pregnancy Childbirth.

[21] Joseph Davey DL, Dovel K, Cleary S (2022). Stepped care to optimize pre-exposure prophylaxis (PrEP) effectiveness in pregnant and postpartum women (SCOPE-PP) in South Africa: a randomized control trial. BMC Public Health.

[22] Iqbal M, Wasim T, AlQahtani SA (2024). The thematic analysis of barriers to immediate post-partum long-acting reversible contraception. Healthcare (Basel).

[23] Mapunda B, August F, Mwakawanga D (2022). Prevalence and barriers to male involvement in antenatal care in Dar es Salaam, Tanzania: A facility-based mixed-methods study. PLoS One.

[24] Humphrey J, Alera M, Kipchumba B (2021). A qualitative study of the barriers and enhancers to retention in care for pregnant and postpartum women living with HIV. PLOS Glob Public Health.

[25] Rafii F, Rahimparvar SF, Mehrdad N, Keramat A (2017). Barriers to postpartum screening for type 2 diabetes: a qualitative study of women with previous gestational diabetes. Pan Afr Med J.

[26] Bains S, Skråning S, Sundby J (2021). Challenges and barriers to optimal maternity care for recently migrated women - a mixed-method study in Norway. BMC Pregnancy Childbirth.

[27] Moniz MH, Dalton VK, Smith RD (2022). Feasibility and acceptability of a toolkit-based process to implement patient-centered, immediate postpartum long-acting reversible contraception services. Am J Obstet Gynecol.

[28] Clouse K, Motlhatlhedi M, Bonnet K (2018). "I just wish that everything is in one place": facilitators and barriers to continuity of care among HIV-positive, postpartum women with a non-communicable disease in South Africa. AIDS Care.

[29] Cartwright PS, McLaughlin FJ, Martinez AM (1993). Teenagers' perceptions of barriers to prenatal care. South Med J.

[30] Yue J, Liu J, Williams S (2020). Barriers and facilitators of kangaroo mother care adoption in five Chinese hospitals: a qualitative study. BMC Public Health.

[31] Watt MH, Cichowitz C, Kisigo G (2019). Predictors of postpartum HIV care engagement for women enrolled in prevention of mother-to-child transmission (PMTCT) programs in Tanzania. AIDS Care.

[32] Hofmann G, Hampanda K, Harrison MS (2022). Virtual prenatal and postpartum care acceptability among maternity care providers. Matern Child Health J.

[33] Dibaba Degefa B, Feyisa GT, Dinagde DD (2023). Post-natal care: a vital chance to save mothers and infants! Exploring barriers and factors associated with it: a mixed study. Front Glob Womens Health.

[34] Usmanova G, Gresh A, Cohen MA (2020). Acceptability and barriers to use of the ASMAN provider-facing electronic platform for peripartum care in public facilities in Madhya Pradesh and Rajasthan, India: a qualitative study using the technology acceptance Model-3. Int J Environ Res Public Health.

[35] Poon Z, Tan NC (2022). A qualitative research study of primary care physicians' views of telehealth in delivering postnatal care to women. BMC Prim Care.

[36] Patel RC, Oyaro P, Thomas KK (2023). Impact of point-of-care HIV viral load and targeted drug resistance mutation testing on viral suppression among Kenyan pregnant and postpartum women: results from a prospective cohort study (Opt4Mamas). J Int AIDS Soc.

[37] Endres K, Razavi N, Tian Z (2023). A retrospective analysis of complications associated with postpartum hemorrhage up to 1 year postpartum in mothers with and without a pre-existing mental health diagnosis. Womens Health (Lond).

[38] Udenigwe O, Okonofua FE, Ntoimo LF (2021). Perspectives of policymakers and health providers on barriers and facilitators to skilled pregnancy care: findings from a qualitative study in rural Nigeria. BMC Pregnancy Childbirth.

[39] Azizi Z, Adedinsewo D, Rodriguez F (2023). Leveraging digital health to improve the cardiovascular health of women. Curr Cardiovasc Risk Rep.

[40] Buchberg MK, Fletcher FE, Vidrine DJ (2015). A mixed-methods approach to understanding barriers to postpartum retention in care among low-income, HIV-infected women. AIDS Patient Care STDS.

[41] Chan SE, Nowik CM, Pudwell J, Smith GN (2021). Standardized postpartum follow-up for women with pregnancy complications: barriers to access and perceptions of maternal cardiovascular risk. J Obstet Gynaecol Can.

[42] Bujimalla PV, Kenne KA, Steffen HA (2024). Effects of rurality and distance to care on perinatal outcomes over a 1-year period during the COVID-19 pandemic. J Rural Health.

